# IKBA phosphorylation governs human sperm motility through ACC-mediated fatty acid beta-oxidation

**DOI:** 10.1038/s42003-023-04693-6

**Published:** 2023-03-25

**Authors:** Yanquan Li, Youwei Hu, Zhengquan Wang, Tingting Lu, Yiting Yang, Hua Diao, Xiaoguo Zheng, Chong Xie, Ping Zhang, Xuelian Zhang, Yuchuan Zhou

**Affiliations:** 1 International Peace Maternity and Child Health Hospital, School of Medicine, Shanghai Jiao Tong University, Shanghai, China; 2 Shanghai Key Laboratory of Embryo Original Diseases, Shanghai, China; 3 State Key Laboratory of Genetic Engineering, School of Life Science, Fudan University, Shanghai, China; 4 NPFPC Key Laboratory of Contraceptives and Devices, Shanghai Institute for Biomedical and Pharmaceutical Technologies, 200032 Shanghai, China

**Keywords:** Lipid signalling, Phosphorylation

## Abstract

The nuclear factor-κB (NF-κB) signaling pathway regulates specific immunological responses and controls a wide range of physiological processes. NF-κB inhibitor alpha (IKBA) is an NF-κB inhibitory mediator in the cytoplasm that modulates the nuclear translocation and DNA binding activities of NF-κB proteins. However, whether the upstream cascade of the canonical NF-κB signaling pathway has physiological roles independent of IKBA-mediated transcriptional activation remains unclear. Herein we investigated the function of IKBA in mature sperm in which transcriptional and translational events do not occur. IKBA was highly expressed in human sperm. The repression of IKBA phosphorylation by its inhibitor Bay117082 markedly enhanced sperm motility. On the contrary, lipopolysaccharide-stimulated IKBA phosphorylation significantly decreased sperm motility. Nevertheless, Bay117082 treatment did not affect the motility of IKBA-knockout sperm. Further, untargeted metabolomic analysis and pharmacological blocking assays revealed that the Bay117082-induced increase in sperm motility was attributable to fatty acid β-oxidation (FAO) enhancement. In addition, we found that IKBA phosphorylation inhibition resulted in a significant reduction of acetyl-CoA carboxylase levels in the FAO metabolic pathway. Our findings indicate that IKBA-mediated signaling orchestrates sperm motility program and improves our understanding of transcription-independent NF-κB signaling pathway in cells.

## Abstract (web summary)

NF-κB inhibitor alpha (IKBA) signaling in mature sperm drives sperm motility and controls fatty acid b-oxidation metabolism.

## Introduction

The nuclear factor-κB (NF-κB) signaling pathway plays a pivotal role in immune, inflammatory, and stress responses, as well as in cell differentiation, proliferation, survival, and apoptosis^1,2^. The NF-κB inhibitor alpha (IKBA) plays a key role in this canonical pathway maintaining the inactive state of the NF-κB complex in the cytoplasm of most nucleated cells. When cells are stimulated by lipopolysaccharide (LPS) or tumor necrosis factor, IKBA is phosphorylated, ubiquitinated, and degraded by a proteosome-dependent pathway; consequently, the NF-κB complex dissociates and translocates to the nucleus where it then regulates downstream target genes^3–5^. This regulatory process mediated by IKBA and its phosphorylation is well characterized and widely accepted to be primarily involved in nuclear transcriptional response^6,7^. Yet, it has recently been shown that there appears to be a non-genomic pathway of NF-κB signaling molecules in anucleated cells^8–11^. Most members of NF-κB pathway proteins are expressed in platelets which are sensitive to NF-κB inhibitors^9,12–14^. Erythrocytes contain NF-κB and IKBA proteins and can be induced to programmed cell death by Bay117082, an irreversible inhibitor of IKBA phosphorylation that interfering with NF-κB-dependent signaling^8,15^. In addition, some studies on the interaction between NF-κB members and mitochondrial proteins have confirmed the non-classical function of the NF-κB signaling pathway in cell activities^16–18^. Despite these new developments, the non-genomic mechanism of NF-κB signaling regulation remains unclear.

Sperm are highly differentiated terminal cells with tightly condensed chromosomes and in which no transcription and translation occur^19^. Their functional transformation entirely relies on changes in protein composition and a complex array of post-translational modifications. The transcription-dependent effects of NF-κB signaling are nonexistent in spermatozoa; thus, they are ideal for elucidating the non-classical mechanism of the NF-κB pathway. Motility is a characteristic function of sperm. Sperm motility generates enough force to free the shackle sperm cell from the oviductal reservoir. Hyperactivation causes them to penetrate cumulus matrix and is a must for penetrating the oocyte pellucida and consequently achieving fertilization^20–22^. Sperm motility is highly dependent on ATP production. Although glycolysis has been suggested to be the primary energy source for mammalian spermatozoa motility^23–25^, sperm of many species, including humans, can reportedly remain motile for long periods in sugar-free media^26,27^. This suggests that energy sources other than glycolysis help sperm maintain their motility. Mitochondrial fatty acid β-oxidation (FAO), for which exogenous fatty acids are the main source of energy, is an active regulator of bovine and boar sperm motility^28–30^. Some enzymes in FAO reaction are evidently expressed in sperm. Some studies found that the inhibition of FAO by etomoxir resulted in a significant reduction in sperm motility^30–33^. According to proteomics- and metabolomics-based studies, human sperm cells contain enzymes involved in lipid metabolism, including those associated with mitochondrial FAO for energy production^31,34^. Thus, it is debatable whether glycolysis and/or oxidative phosphorylation is the only or the major supplier of ATP needed for human sperm motility. Further, it not fully understood how sperm maneuver between these processes for energy production^26,27,35,36^. In addition, the characteristics of FAO-related modulators in human spermatozoa is unknown.

Herein, we investigated the role of IKBA phosphorylation in sperm motility and elucidated the underlying molecular mechanism via metabolic tracing, pharmacological assessments, and IKBA-knockout mouse model.

## Results

### Expression of canonical NF-κB components in human sperm

**Fig. 1:**
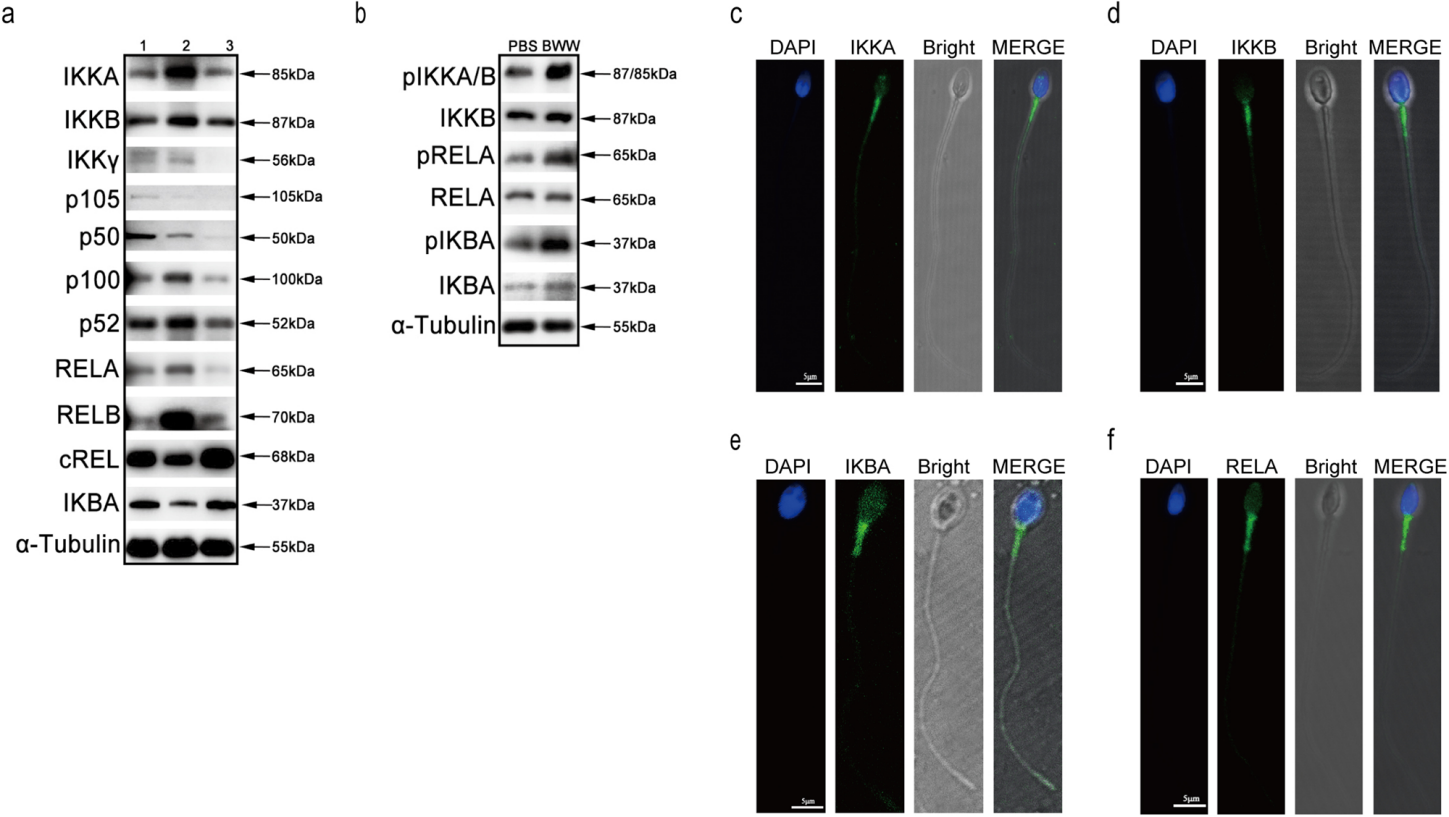
Expression and localization of canonical NF-κB proteins in human sperm. **a** Expression of IKKA, IKKB, IKKγ, p105/p50 (NF-κB1), p100/p52 (NF-κB2), RELA (p65), RELB, cREL, and IKBA in human sperm. α-Tubulin served as the loading control. Numbers represent samples of different individuals. **b** Phosphorylation states of IKKA/B, RELA and IKBA proteins in human sperm in PBS and BWW media. α-Tubulin served as the loading control. **c**–**f** Localization of IKKA (**c**), IKKB (**d**), IKBA (**e**) and RELA (**f**) proteins in human sperm. DAPI (blue) labeled the nuclei; IKKA, IKKB, IKBA, and RELA were stained with AlexaFluor488 (green) separately. Fluorescent images were merged with bright-field images (Bright) shown in the MERGE panels. Scale bar: 5 μm.

To investigate the expression of NF-κB components in human sperm, we performed western blotting and indirect immunofluorescence assays. We found the presence of most NF-κB proteins, including IKKA, IKKB, IKKγ, p105/50, p100/52, RELA/B, cREL, and IKBA in human sperm (Fig. 1a). The phosphorylation state of IKKA/B, RELA, and IKBA constitutively existed in these cells, and their phosphorylation was further enhanced when sperm were incubated in Biggers–Whitten–Whittingham (BWW) media (Fig. 1b). We selected several proteins in NF-κB components and observed their localization. IKKA, IKKB, IKBA, and RELA subunits were found to be distributed in the midpiece of sperm (Fig. 1c–f), where mitochondria are localized. The expression and distribution of NF-κB proteins suggested their involvement in the regulation of human sperm functions.

### IKBA phosphorylation regulates sperm motility

**Fig. 2:**
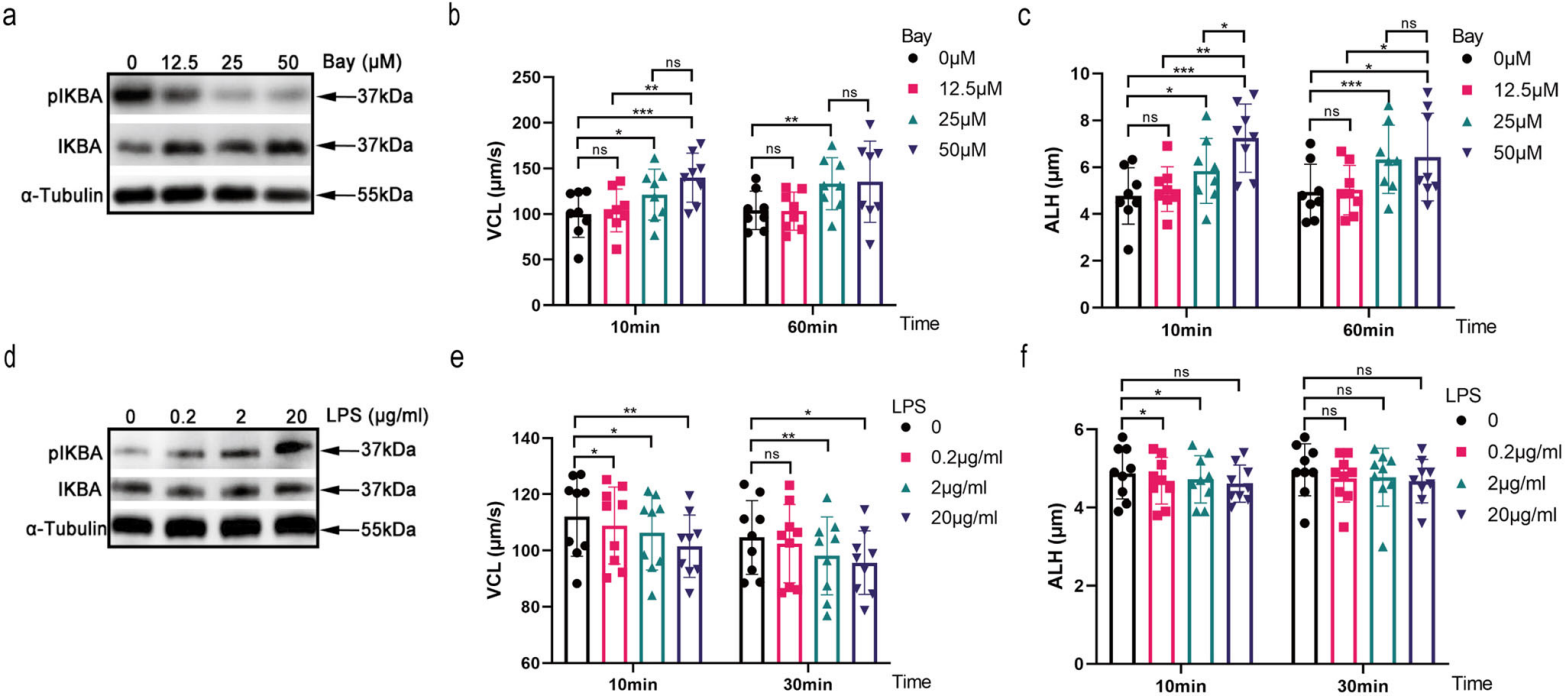
IKBA phosphorylation regulates sperm motility. **a** Western blotting of IKBA and pIKBA in human sperm treated with different concentrations of Bay117082 (0, 12.5, 25 and 50 μM) for 10 min. α-Tubulin served as the loading control. **b**, **c** Dose-dependent effect of Bay117082 on sperm motility. Sperm were incubated with 0, 12.5, 25, and 50 μM Bay117082 for 10 and 60 mins in BWW media. The sperm motility parameters VCL (**b**) and ALH (**c**) were measured by CASA. Values represent mean ± SEM (*n* = 8). *p < 0.05; **p < 0.01; ***p < 0.001, as compared with the corresponding control (0 µM). ns, no significance. **d** Western blotting of IKBA and pIKBA in human sperm treated with different concentrations of LPS (0, 0.2, 2 and 20 μg/mL) for 10 min. α-Tubulin served as the loading control. **e**, **f** Dose-dependent effects of LPS on sperm motility. Sperm were incubated with final concentrations of LPS at 0, 0.2, 2, and 20 μg/mL for 10 and 30 mins. VCL (**e**) and ALH (**f**) were examined by CASA. Values represent mean ± SEM (*n* = 9). *p < 0.05; **p < 0.01, as compared with the corresponding control (0 μg/mL). ns, no significance.

IKBA phosphorylation is the core event in NF-κB signaling activation. To investigate the functional roles of IKBA proteins, we assessed the effect of Bay117082, an IKBA phosphorylation inhibitor, on human sperm motility. Bay117082 was found to inhibit IKBA phosphorylation (Fig. 2a). Correspondingly, Bay117082 strongly enhanced the curvilinear velocity (VCL) and amplitude of lateral head displacement (ALH) of human sperm in a dose-dependent but not in a time-dependent manner (Fig. 2b, c). At 25 μM, the enhancement of curvilinear movement was still effective for 60 min. No difference in VCL were detected between 25 μM and 50 μM at 10 and 60 mins (Fig. 2b). Therefore, we selected 25 μM Bay117082 as the ideal concentration and 10 min as the ideal treatment duration for subsequent experiments, unless otherwise specified. To verify the role of IKBA phosphorylation activation in motility, sperm were treated with LPS, a stimulator of IKBA phosphorylation. LPS activated IKBA phosphorylation (Fig. 2d) and significantly inhibited sperm motility (Fig. 2e, f). Collectively, these findings suggest that IKBA phosphorylation regulates parameters related to human sperm hyperactivation.

### IKBA phosphorylation alters sperm swimming patterns from forward to curvilinear movement

**Fig. 3:**
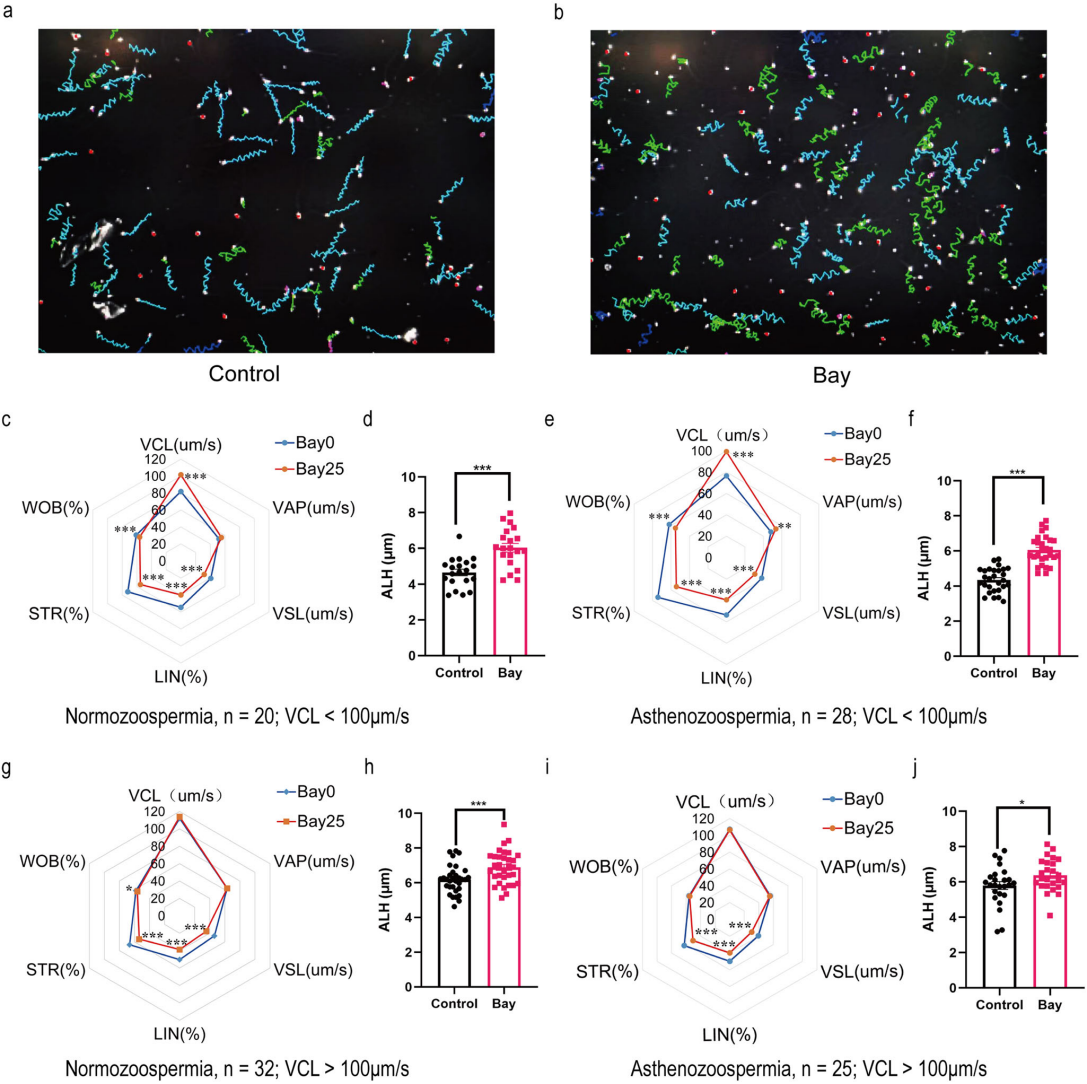
IκBα phosphorylation alters sperm swimming patterns from forward to curvilinear movement. **a**, **b** Sperm trajectories before (**a**) and after (**b**) treatment with 25 μM Bay117082, as recorded by CASA. **c**–**j** Changes in sperm motility parameters after treatment with 25 μM Bay117082 for 10 min. VCL, VAP, VSL, LIN, STR and WOB are presented in radar charts. ALH is shown in bar graphs. **c**–**f** Bay117082-induced changes in parameters related to the motility of normozoospermic (**c**, **d**) and asthenozoospermic (**e**, **f**) sperm, with basal VCL ≤ 100 μm/s. **g**–**j** Bay117082-induced changes in parameters related to the motility of normozoospermic (**g**, **h**) and asthenozoospermic (**i**, **j**) sperm with basal VCL > 100 μm/s. *p < 0.05; **p < 0.01; ***p < 0.001, as compared with the control (0 μM).

Hyperactivated sperm mainly display low straight-line velocity (VSL) or high VCL and ALH. We investigated Bay117082-induced changes in sperm motility pattern. Trajectories of control (Fig. 3a) and Bay117082-treated (Fig. 3b) sperm recorded by computer-assisted sperm analysis (CASA) showed an obvious change from long linear trajectories to short wide wavy trajectories. Next, we analyzed the linearity and curvilinearity of sperm movement and found that regardless of normozoospermia or asthenozoospermia, when the basal VCL value was ≤ 100 μm/s, Bay117082 significantly increased the VCL of normozoospermic sperm from 81.7570 ± 7.7440 μm/s to 101.4965 ± 18.4700 μm/s (*p* < 0.001) and VCL of asthenozoospermic sperm from 76.5007 ± 10.3311 μm/s to 99.2404 ± 13.5237 μm/s (*p* < 0.001) (Fig. 3c, e). However, when basal VCL was >100 μm/s, the VCL of both normozoospermic (111.9934 ± 13.3063 μm/s vs 113.9119 ± 15.2855, *p* = 0.245) and asthenozoospermic (107.1364 ± 14.2916 μm/s vs 106.4524 ± 14.3532, *p* = 0.741) sperm did not show further increase on Bay117082 treatment (Fig. 3g, i). Under all conditions, Bay117082 significantly increased ALH (Fig. 3d, f, h, j). In contrast, Bay117082 reduced VSL, linearity (LIN) and straightness (STR) (Fig. 3c, e, g, i), which are forward movement-related parameters. Altogether, we observed that Bay117082 altered sperm swimming patterns from forward to curvilinear movement (see Supplementary Videos 1–4 for corresponding videos).

### Bay117082-induced enhancement of sperm motility involves fatty acids consumption for energy production

**Fig. 4:**
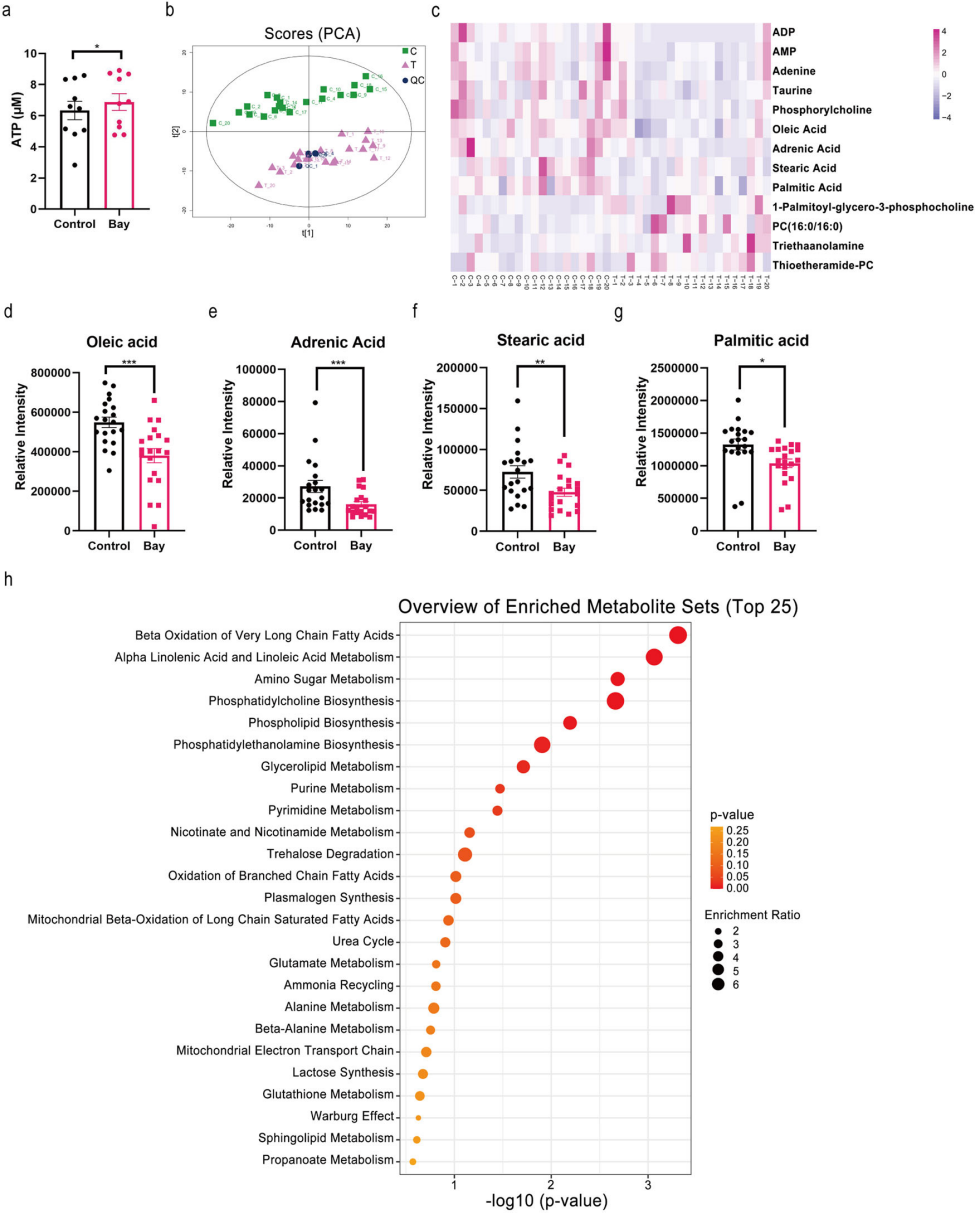
Bay117082-induced sperm motility enhancement consumes fatty acids as the energy source. **a** Effects of 25 μM Bay117082 on sperm ATP level. Values represent mean ± SEM (*n* = 10), *p < 0.05, as compared with the control (0 μM). **b** Principal component (PCA) analysis of untargeted metabolomics data obtained from Bay117082-treated sperm and control sperm. Each dot on plot represents an individual sample (*n* = 20). Green dots represented the control group (C); purple dots represent the Bay117082-treated group (T); blue dots represent the quality control samples. **c** Heatmap of significantly different metabolites in the control (C) and Bay117082-treated (T) group. The variable importance in the projection (VIP) value of each variable in the orthogonal partial least squares discriminant analysis (OPLS-DA) model was calculated. Significance was determined using paired Student’s *t* test. VIP > 1 and *p* < 0.05 were applied to identify statistically significant differential metabolites. The *x*-axis represents comparative changes in each sperm sample before (C1–C20) and after (T1–T20) Bay117082 treatment. Significantly different metabolites were labeled on the *y*-axis. **d**–**g** Bay117082-induced changes in oleic acid (**d**), adrenic acid (**e**), stearic acid (**f**), and palmitic acid (**g**) in sperm. Values represent mean ± SEM (*n* = 20), *p < 0.05; **p < 0.01; ***p < 0.001, as compared with the control (0 μM). **h** Metabolite Set Enrichment Analysis based on the Small Molecule Pathway Database. The top 25 of enriched pathways are shown.

Sperm motility depends on energy supply. Herein on measuring changes in ATP content in sperm without or with Bay117082 treatment, we found that Bay117082 treatment significantly increased ATP levels in sperm (Fig. 4a). Next, we equally divided sperm into control group (C) and treated group (T), followed incubation for 10 min without or with 25 μM Bay117082, respectively. Sperm were subsequently analyzed via untargeted metabolomics by liquid chromatography-mass spectrometry (LC-MS/MS) analysis. Principal component analysis indicated that the treated and control groups could be significantly grouped (Fig. 4b). As evident from the heatmap shown in Fig. 4c, relative to the control group, the level of four metabolites showed significant up-regulation [Thioetheramide-PC, 1-Palmitoyl-sn-glycero-3-phosphocholine, PC (16:0/16:0), and Triethanolamine] and those of nine showed down-regulation [palmitic acid, phosphorylcholine, oleic acid, stearic acid, adrenic acid, adenosine monophosphate (AMP), adenosine diphosphate (ADP), adenine, and taurine] in the treated group. Further, in the treated group, the levels of several fatty acids (Fig. 4d–g) including oleic acid (C_18_H_34_O_2_), adrenic acid (C_22_H_36_O_2_), stearic acid (C_18_H_36_O_2_) and palmitic acid (C_16_H_32_O_2_), were markedly downregulated, suggesting the consumption of long-chain fatty acids on treatment with Bay117082. Furthermore, metabolite set enrichment analysis that based on the small molecule pathway database highlighted several metabolic pathways of sperm (Fig. 4h). β-oxidation of long-chain fatty acids was significantly enriched, indicating its important role in sperm motility alteration. These results suggest that Bay117082-activated sperm motility is closely related to FAO.

### Sperm motility enhancement by Bay117082 is not dependent on glucose metabolism

**Fig. 5:**
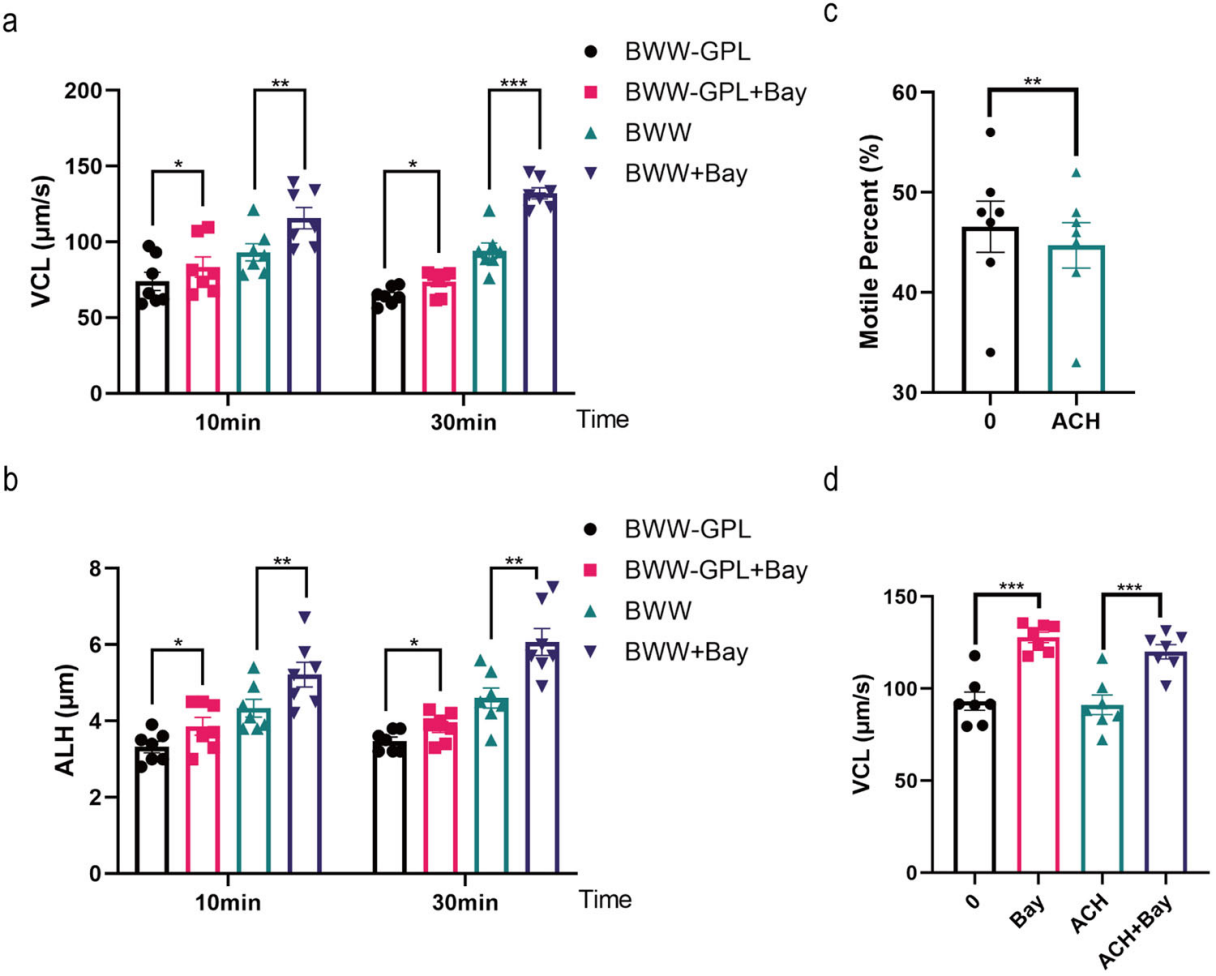
Bay117082 promotes sperm motility independently of glucose metabolism. **a**, **b** Changes in VCL and ALH of sperm treated with 25 μM Bay117082 in BWW-GPL or BWW media for 10 and 30 mins. BWW-GPL: BWW media lacking glucose (G), pyruvate (P) and lactic acid (L). Values represent mean ± SEM (*n* = 7), *p < 0.05, **p < 0.01, ***p < 0.001, as compared with the corresponding control (0 μM) in each medium. **c** Changes in motile percent of sperm incubated with 500 μM α-chlorohydrin (ACH) for 1 h. Values represent mean ± SEM (*n* = 7), **p < 0.01. **d** Changes in VCL of sperm treated with 25 μM Bay117082 in the absence or presence of 500 μM ACH for 1 h. Values represent mean ± SEM (*n* = 7), ***p < 0.001, as compared with the corresponding control.

In view of previous studies, glycolysis is the primary supplier of ATP to human sperm^24^. We investigated the effects of Bay117082 on sperm motility in the absence of glucose, pyruvate, and lactic acid (BWW-GPL). Bay117082 was found to still significantly promote sperm motility in BWW-GPL media (Fig. 5a, b). Furthermore, human sperm were treated with α-chlorohydrin (ACH), an inhibitor of glycolysis, to verify if the effects of Bay117082 on sperm motility were independent on glycolysis. As indicated in Fig. 5c and by a previous study^37^, treatment with 500 μM ACH for 1 h effectively downregulated the percentage of sperm motility. However, sperm motility stimulated by Bay117082 could not be significantly blocked by ACH (Fig. 5d). The results suggest that the promotion of sperm motility by Bay117082 is not mainly dependent on glucose metabolism.

### IKBA phosphorylation inhibition by Bay117082 regulates FAO in human sperm

**Fig. 6:**
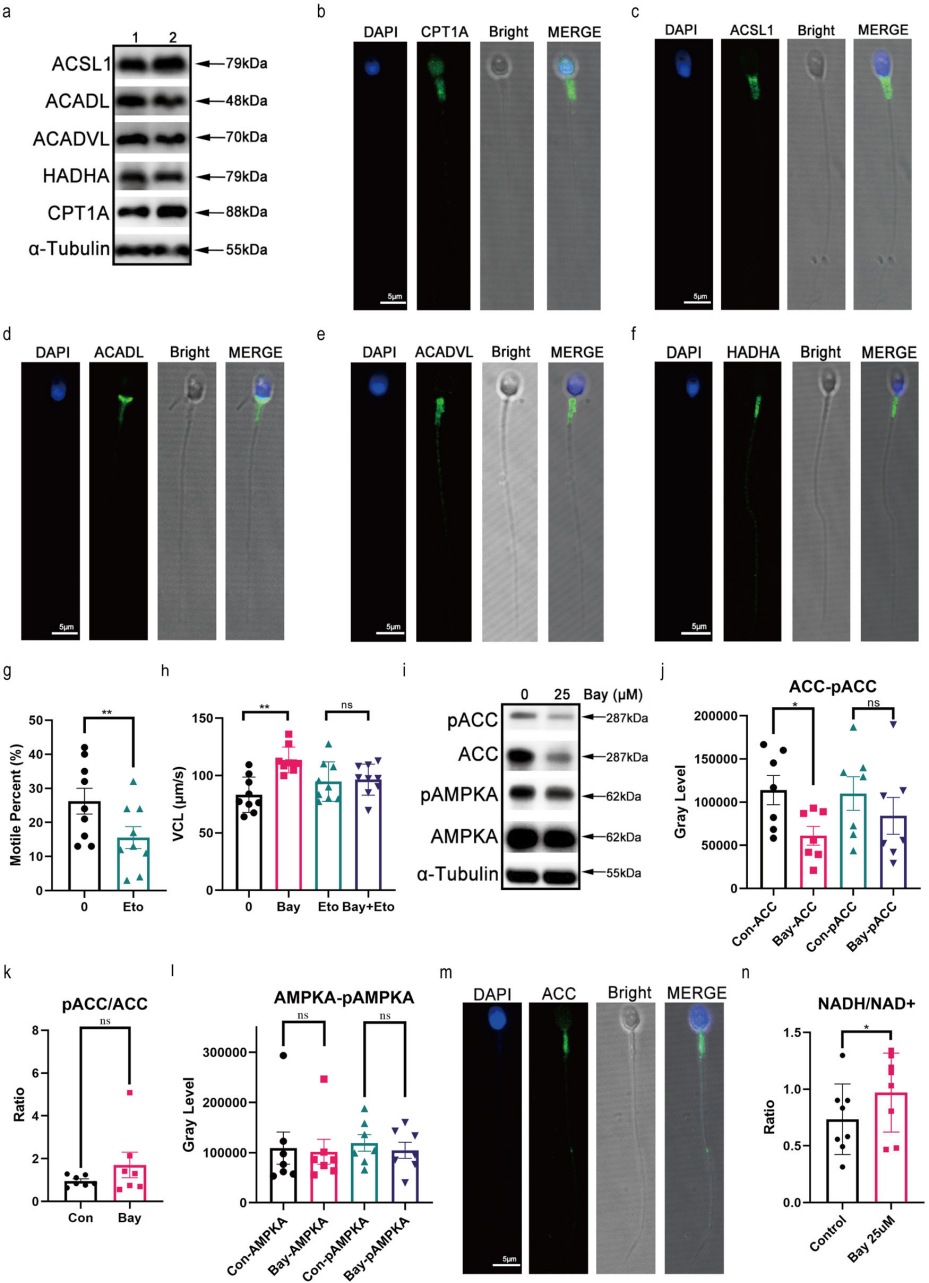
IKBA phosphorylation inhibition by Bay117082 regulates FAO in human sperm. **a** Expression of ACSL1, ACADL, ACADVL, HADHA, and CPT1A in sperm. α-Tubulin was applied to assess protein loading. Numbers represent samples of different individuals. **b**–**f** ACSL1, ACADL, ACADVL, HADHA and CPT1A localization in sperm. DAPI (blue) labeled the nuclei. CPT1A (**b**), ACSL1 (**c**), ACADL (**d**), ACADVL (**e**), and HADHA (**f**) were stained with AlexaFluor488 (green). Images were merged (MERGE) with bright-field images (Bright). Scale bar: 5 μm. **g** Motile percent of sperm treated with etomoxir (400 μM) for 1 h in BWW media. Values represent mean ± SEM (*n* = 9), **p < 0.01, compared with the corresponding control (0 μM). **h** Changes in VCL of sperm treated with 25 μM Bay117082 in the absence or presence of etomoxir (400 μM) for 1 h. Values represent mean ± SEM (*n* = 9), **p < 0.01, compared with the corresponding control. ns, no significance. **i**–**l** Western blotting showing AMPKA, ACC, pAMPKA, and pACC levels (**i**) in sperm treated with or without Bay117082 at 25 μM for 10 min. Gray-scale value analysis of ACC and pACC (**j**), pACC/ACC (**k**), as well as AMPKA and pAMPKA (**l**). *n* = 7, *p < 0.05, compared with the corresponding control (0 μM). ns, no significance. α-Tubulin was applied to assess protein loading. **m** Indirect immunofluorescence showing ACC localization in human sperm. DAPI (blue) labeled the nuclei; ACC was stained with Alexa Fluor 488 (green). Images were merged (MERGE) with bright-field images (Bright). Scale bar: 5 μm. **n** Changes in NADH/NAD^+^ levels in sperm treated with or without Bay117082 in BWW-GPL media. Values represent mean ± SEM (*n* = 8), *p < 0.05.

To validate Bay117082-induced utilization of FAO, we examined the expression of some key FAO-associated proteins or enzymes. Carnitine palmitoyl transferase 1A (CPT1A), acyl-CoA synthetase long-chain family member 1 (ACSL1), long-chain specific acyl-CoA dehydrogenase (ACADL), very long-chain specific acyl-CoA dehydrogenase (ACADVL) and hydratase subunit A (HADHA) were expressed in sperm and found in the midpiece of sperm tail, where mitochondria are located (Fig. 6a–f). CPT1A is a rate-limiting enzyme of FAO and transports activated long-chain fatty acids from the cytoplasm into mitochondria. Etomoxir, an inhibitor of CPT1A, can reportedly significantly decrease human sperm motility^31^. Herein we treated sperm with etomoxir and the same result was obtained (Fig. 6g). Furthermore, etomoxir significantly abolished sperm motility enhancement induced by Bay117082 (Fig. 6h). The AMP-activated protein kinase (AMPK)-acetyl-CoA carboxylase (ACC)-CPT1A axis tightly regulates long-chain fatty acid oxidation in mitochondria^38^. We investigated the state of AMPK and ACC proteins as well as their phosphorylation before and after Bay117082 treatment. On Bay117082 treatment, ACC protein content showed a significant decrease in sperm, whereas the level of pACC/ACC showed an upward trend (Fig. 6i–k). There was no significant change in AMPKA, the catalytic subunit of AMPK, and its phosphorylation level in sperm before and after Bay117082 treatment (Fig. 6i, l). We also found that ACC was localized to the mitochondria in sperm midpiece (Fig. 6m), similar to the localization of IKBA and key FAO enzymes. In addition, Bay117082 treatment elevated the content of NADH/NAD^+^, the products of FAO, in BWW-GPL media (Fig. 6n). These results suggest that Bay117082 activates FAO to release energy and enhance movement of sperm.

### IKBA^-/-^ mouse sperm lost their response to Bay117082

**Fig. 7:**
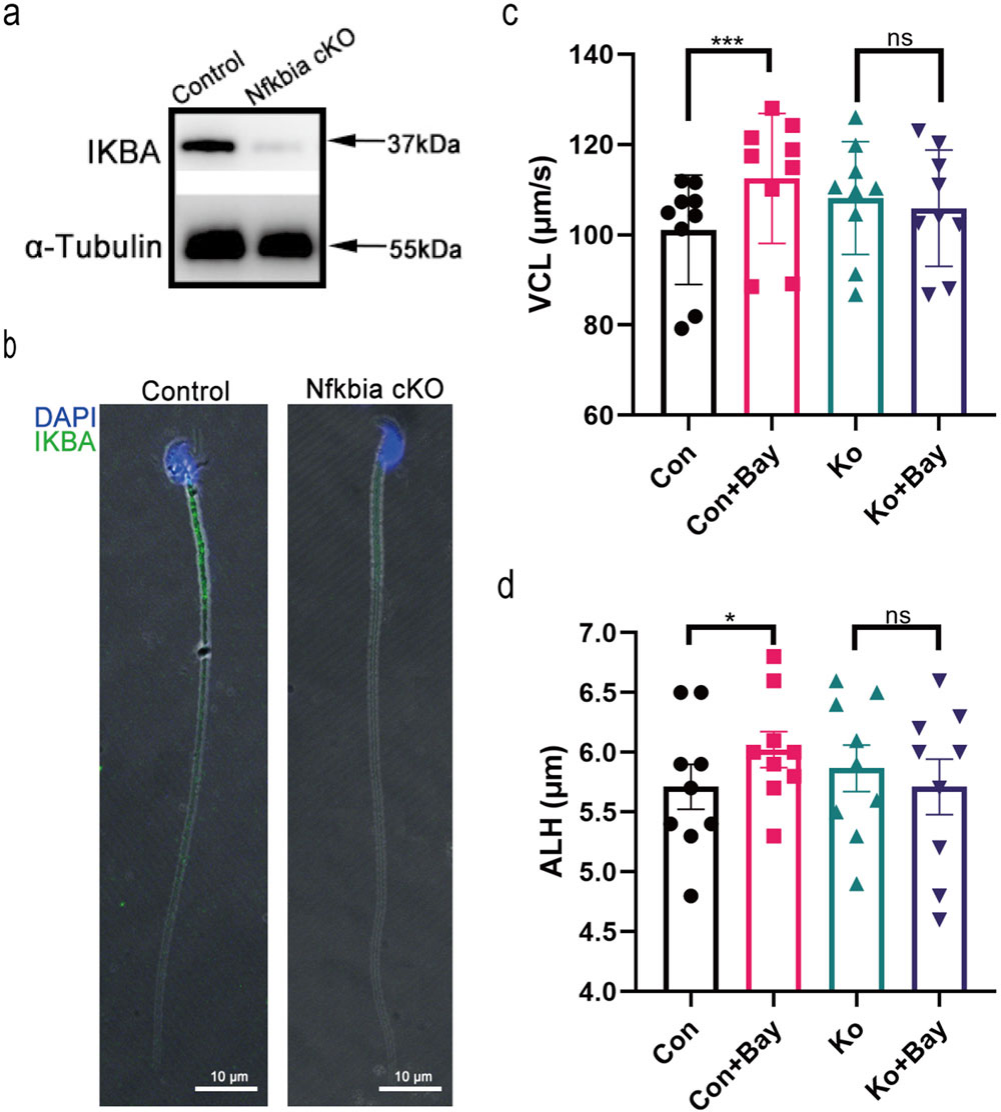
IKBA-/- mouse sperm showed no response to Bay117082. **a** Expression level of IKBA in control and Nfkbia cKO mice sperm. α-Tubulin was applied to assess protein loading. **b** Distribution of IKBA in sperm of control mice and Nfkbia cKO mice. DAPI (blue) labeled the nuclei, and IKBA was stained with Alexa Fluor 488 (green). Images were merged (MERGE) with bright-field images (Bright). Scale bar: 10 μm. **c**, **d** Changes in VCL and ALH of control and Nfkbia cKO mice sperm treated with or without Bay117082 for 10 min. Values represent mean ± SEM (*n* = 9), *p < 0.05, ***p < 0.001, compared with the corresponding control (0 μM). ns, no significance.

Bay117082 is a broad-spectrum inhibitor with multiple targets^39^. To exclude the nonspecific role of Bay117082 in sperm motility regulation, we generated an IKBA conditional knockout mouse (IKBA cKO) model based on the CRISPR-Cas9 and Cre-/LoxP technologies (*Nfkbia*^*f/f*^; *Stra8-Cre*) (Supplementary Fig. 1a, b). Genotyping PCR was performed to assess the genotype of mice (Supplementary Fig. 1c, d). Western blotting and immunofluorescence analysis indicated the deletion of IKBA in (*Nfkbia*^*f/f*^; *Stra8-Cre* + ) mouse (IKBA^-/-^) sperm (Fig. 7a, b). Motility parameters showed no statistically significant difference between IKBA^-/-^ and control sperm (Supplementary Fig. 1e). However, IKBA^-/-^ sperm lost their response to Bay117082 (Fig. 7c, d). Altogether, these findings indicated that Bay117082 regulates sperm motility by specifically targeting IKBA phosphorylation.

## Discussion

Both activated and hyperactivated sperm motility are pivotal for fertilization^20^. Sperm motility requires large amounts of ATP. According to previous studies^24,25,40^, carbohydrates are the main source of energy for sperm motility. In this study, FAO was found to play a chief role in generating energy needed for human sperm motility. Moreover, we believe that Bay117082 can be used to enhance human sperm motility. Our results provide novel insights into the molecular mechanisms underlying the regulation of energy needed for sperm motility and lay a foundation for the development of Bay117082 as a potential therapeutic to tackle male infertility. We also found that almost all known NF-κB family members were expressed in human sperm. Besides, IKBA phosphorylation, a modification of specific NF-κB proteins, was sensitive to stimulation and served functions other than the regulation of transcription. We report a novel and ideal cell model to study the molecular mechanism of the NF-κB signaling pathway in the absence of NF-κB translocation to the nucleus. Our findings indicate that IKBA-mediated signaling orchestrates a transcription-independent sperm motility system and invite to rethink and evaluate non-classic NF-κB signaling in somatic cells.

Mammalian sperm constantly metabolize extracellular or/and intracellular energy substrates to generate ATP so as to maintain their motility for prolonged periods. ATP is mainly derived from glycolysis and mitochondrial oxidative phosphorylation with glucose, pyruvate, and lactic acid serving as the substrates. However, according to recent studies, sperm of different species employ diverse metabolic strategies to alter their motility based on physiological or pathological stimuli^41^. For instance, dog sperm are capable of synthesizing and degrading glycogen, which is transformed into glucose-6-phosphate and then via glycolysis, energy is supplied to sperm^42,43^. Ketone body catabolism also reportedly contributes to ATP production to support mouse sperm motility^44^. The hydrolysis of phospholipids to produce glycerol, which can enter the glycolytic pathway through the sequential conversion to glycerol-3-phosphate and dihydroxyacetone phosphate, is evidently correlated to pig and bull sperm motility^41^. It has been previously suggested that fatty acids are the main source of energy and improve bovine and boar sperm motility^28–30^. Furthermore, dolphin sperm motility appears to depend almost exclusively on the oxidation of endogenous fatty acids^45^. An earlier study reported that Slc22a14-mediated FAO is essential for spermatozoa energy generation and motility in mice^33^. In case of human sperm, ATP required for sperm motility is usually believed to be mainly derived from oxidative phosphorylation and glycolysis, and sperm response to changes in energy demand is regulated by glycolytic flux rather than by mitochondrial respiration^24,26,27,41,46^. Nevertheless, it has been found that human sperm can remain motile for quite a long period in the absence of glucose, pyruvate, and lactic acid^26,27,47^, suggesting that besides glycolysis and carbohydrates, other energy sources are available to support spermatozoa motility. Proteomic studies have suggested that FAO contributes to ATP generation and human sperm motility. Enzymes associated with mitochondrial β-oxidation have been identified in sperm tail, and etomoxir, a fatty acid oxidation inhibitor, was reported to significantly decrease sperm motility^31^. Our results indicated FAO can be mobilized by IKBA phosphorylation to provide energy to human sperm and regulate their motility, supporting the view that in addition to carbohydrates (glucose, pyruvate, and lactic acid), lipids are an important source of energy for human sperm motility. This implies that most mammalian spermatozoa employ a versatile metabolic strategy to maintain their motility. Future studies should focus on comprehensively investigating these strategies under specific circumstances. It is notable that the inhibition of IKBA phosphorylation by Bay117082 accelerated sperm motility within 10 min. One molecule of glucose generates only two ATP molecules if incompletely metabolized by glycolysis and lactic acid fermentation, and there are 32-26 ATP molecules are produced when one glucose molecule is completely aerobically combusted^24^. In the other hand, β-oxidation of one molecule 16 C fatty acid, such as palmitoyl-CoA, generates 106 ATP molecules^48^. Therefore, the activation of β-oxidation can quickly improve sperm motility. The lipid composition of spermatozoa shows certain specific characteristics, such as a higher proportion of neutral lipids^49^. In asthenozoospermic men, oleic acid and palmitic acid levels are relatively higher, signifying a metabolic disorder of the sperm lipids^50,51^. We believe that in human sperm, FAO is an important backup strategy for the regulation of hyperactivation motility.

Initially, Bay117082 was identified to be a specific inhibitor of NF-κB kinase (IKK) in the NF-κB signaling pathway; it was found to selectively inhibit cytokine-induced IKBA phosphorylation to exert anti-inflammatory activity in vivo^52^. Later, it was demonstrated that Bay117082 was a broad-spectrum inhibitor with anti-inflammatory activity against multiple targets^39,53,54^. In addition, Bay117082 displayed other pharmacological activities that include anticancer and neuroprotective effects^39^. A previous report demonstrated Bay117082 to be an irreversible inhibitor of protein tyrosine phosphatases (PTPs), which specifically dephosphorylate tyrosine residues in proteins to modify their tyrosine phosphorylation status in cells and evidently enhance protein phosphorylation^55^. In sperm, the enhancement of protein tyrosine phosphorylation is the indicator of capacitation which is accompanied by hyperactivation. PTPs activity has a positive role in the regulation of mammalian sperm motility and protein tyrosine phosphorylation^56^. Herein we ruled out the possibility that Bay117082 regulates sperm motility by inhibiting PTPs. In bull and mouse sperm, hyperactivated motility is not always positively correlated to an increase in capacitation-associated protein tyrosine phosphorylation^57,58^. Further, there exists no significant relationship between human sperm motility and tyrosine phosphorylation^59^. Previous studies have reported that tyrosine phosphorylation shows a slow and gradual time-dependent increase during sperm capacitation^60–62^. In this study, Bay117082 induced an obvious increase in protein tyrosine phosphorylation at 60 min (Supplementary Fig. 2a). Nevertheless, the effect of Bay117082 on sperm movement was rapid: it significantly accelerated sperm motility in 10 min or even as fast as 2 min in our experiments. A significant increase in tyrosine phosphorylation by Bay117082 at 1 h did not further enhance sperm motility (Supplementary Fig. 2b and Fig. 2b, c). It has been previously reported that, PTPs activity inhibition reduces the overall velocity of hamster, boar, stallion, dog mouse and human spermatozoa^56,63,64^. While Bay117082 significantly increased sperm motility, it was unable to induce any such change after IKBA gene deletion in our mouse model. This suggests that Bay117082 specifically targets the phosphorylation of IKBA protein. Without a doubt, we cannot completely exclude the influence of other unknown targets of Bay117082 on sperm motility.

As mature sperm lacks complete cytoplasm and most organelles, it is plausible that IKBA serves some function other than the regulation of transcription. The involvement of NF-κB in non-canonical signaling pathways has been previously demonstrated in anucleated cells^8–11^. IKBA evidently interacts with mitochondrial proteins to regulate specific functions^16–18^. In the present study, IKBA protein localized to the mitochondria in sperm midpiece, suggesting that IKBA plays a vital role in supplying energy to sperm. We also confirmed that IKBA phosphorylation was closely related to β-oxidation of mitochondria in human sperm. Altogether, we believe that the IKBA-mediated β-oxidation of activated fatty acids within the mitochondrial matrix represents a key energy source for sperm motility.

**Fig. 8:**
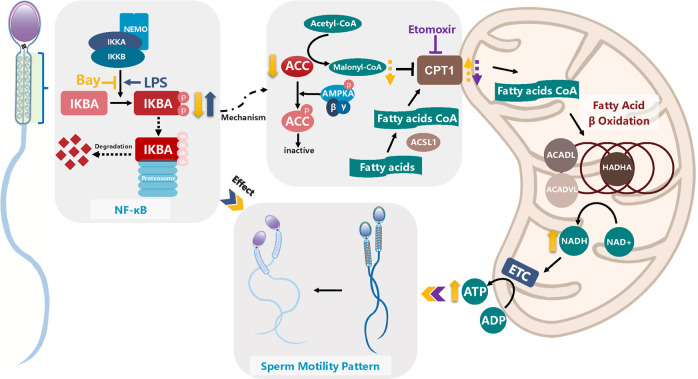
Diagram depicting the regulation mechanism of IKBA phosphorylation in sperm motility. IKBA and IKK subunits located on the midpiece of human sperm. Bay117082 inhibits IKBA phosphorylation and changes the swimming pattern from forward movement to curvilinear movement. On the contrary, LPS induces IKBA phosphorylation and weakens sperm motility. ACC level decrease and pACC/ACC level shows an upward trend when sperm are treated with Bay117082; thus, less acetyl-CoA is catalyzed by ACC to form malonyl-CoA. As malonyl-CoA inactivates CPT1A, the decrease in ACC level or the increase in pACC/ACC level reduces malonyl-CoA production and promote the entry of fatty acids into the mitochondria for catabolism. The key enzymes of FAO, including ACSL1, ACADL, ACADVL, and HADHA, are located on the midpiece of human sperm. NADH levels are elevated in sperm treated with Bay117082, suggesting that increased ATP levels from FAO support increased sperm motility. Etomoxir decreases sperm motility and blocks the action of Bay117082 on sperm motility. Thin black arrows represent known positive physiological process. Thin black dashed arrows indicate a speculative process. Blocked lines represent repressive effect. Thick arrows (up or down) indicate the alteration of substance after treatment with Bay117082 (yellow), LPS (blue), and etomoxir (purple); solid lines represent verified trends and dashed lines represent conjectural trends. The elements “sperm” and “mitochondria” were modified from our previous article^62^.

Lipids are primarily consumed through mitochondrial FAO. Free fatty acids are esterified with CoA and then transferred into the mitochondria matrix for β-oxidation, where they are oxidized into acetyl-CoA and NADH and FADH_2_ are generated. In this process, ACC converts acetyl-CoA to malonyl-CoA. Malonyl-CoA inhibits CPT1, a key enzyme that initiates free fatty acids transportation into mitochondria. Several studies have demonstrated that the AMPK/ACC/CPT1 pathway regulates FAO^38,65^. AMPK inactivates ACC via phosphorylation, preventing malonyl-CoA generation^38,66^. The inhibition of the AMPK/ACC/CPT1A signaling pathway is favorable for the recovery of CPT1 activity and FAO. Our data showed that the inhibition of IKBA phosphorylation by Bay117082 significantly decreased ACC, which explained why Bay117082 promoted β-oxidation. In somatic cells, the activity of ACC can be regulated by transcription and post-transcription as well as degradation according to the metabolic status of cells^67^. No transcription or translation occurs in sperm, and post-translational modification and degradation of proteins are accordingly observed. ACC is reportedly ubiquitylated on interaction with E3 ubiquitin ligase, which subsequently causes ACC degradation by proteasome in adipose tissue^68,69^. E3 ubiquitin ligase is expressed in sperm and performs an essential ubiquitination function^70–72^. Therefore, it is reasonable to speculate that ACC degradation is caused by the ubiquitination degradation pathway. Further studies are warranted to determine whether IKBA directly or indirectly regulates ACC degradation and how this degradation is modulated. Figure 8 shows the potential mechanism via which FAO is activated by Bay117082/IKBA/ACC.

In summary, we discover the non-classical role of IKBA in mature human sperm. Our results indicate that IKBA phosphorylation regulates sperm motility through ACC-mediated FAO. Our data provide novel insights into the regulatory mechanisms associated with supplying energy for human sperm motility. Finally, we believe that Bay117082 can be potentially used to enhance sperm motility and to alleviate male infertility problems, such as asthenozoospermia.

## Methods

### Chemicals/reagents

As recommended by the WHO Laboratory manual for the examination and processing of human semen (fifth edition) and our earlier study^62^, we used BWW media (94.8 mM NaCl, 4.8 mM KCl, 1.7 mM CaCl_2_, 1.2 mM MgSO_4_, 1.2 mM KH_2_PO_4_, 5.5 mM glucose, 13.21 mM sodium lactate, 0.27 mM sodium pyruvate, 25 mM NaHCO_3_, and 3.5 mg/mL BSA) for collecting and cultivating human sperm. The aforementioned reagents were purchased from Sigma-Aldrich (Saint Louis, MO, USA). Bay117082, LPS, α-chlorohydrin, etomoxir and DAPI were also obtained from Sigma-Aldrich. Antibody-related information is shown in Supplementary Table 1.

### Ethical approval

This study, including the process for semen sample collection, was approved by the Ethics Committee on human subjects of International Peace Maternity and Child Health Hospital (GKLW2018-03).

### Sperm sample collection and treatments

Semen samples were collected and treated as previously reported^62^. Briefly, semen was obtained via masturbation from participants with 3-5 days of sexual abstinence who visited outpatient clinics at Reproductive Medicine Center of International Peace Maternity and Child Health Hospital, Shanghai, China, between January 2019 to September 2022. The samples were liquefied at room temperature for at least 30 min before diagnostic semen analysis and scientific research. Subsequently, the samples were subjected to CASA (Hamilton-Thorne, Beverly, MA, USA) to assess sperm motility parameters and classified into normozoospermia or asthenozoospermia according to the WHO laboratory manual for the examination and processing of human semen (fifth edition). We then centrifuged spermatozoa at 500 *g* for 3 min to remove plasma, followed by a BWW medium or PBS buffer wash. Then samples were centrifuged and resuspended in BWW medium or PBS buffer to a final concentration of 10-20 ×10^6^ cells/mL for subsequent experiments. This suspension was divided into several equal parts and treated with Bay117082, LPS, α-chlorohydrin, or etomoxir, either individually or in combination as required. Samples were finally incubated in a 5% CO_2_ incubator at constant temperature of 37 °C for specific time.

### Assessment of sperm motility

Sperm motility and concentration were measured by CASA, as previously described^62^. After treatment with required reagent for specific time, 5 μl of each sample was added to the sperm-counting chamber (Netherland, Leja). Ten microscopic fields of each chamber were analyzed, and at least 200 spermatozoa were evaluated. The following parameters were recorded: percentage of motile sperm (motile percent), average path velocity (VAP, μm/s), progressive velocity (VSL, μm/s), curvilinear line velocity (VCL, μm/s), straightness (STR, %), linearity (LIN, %), amplitude of lateral head (ALH, μm), beat cross frequency (BCF, *Hz*), and wobble (WOB, %). The playback function of the system was used to verify its accuracy.

### Western blotting

Sperm samples were collected and purified. Pellets of spermatozoa were lysed in Laemmli buffer (0.0625 M Tris base, 2% SDS, 10% glycerol, 0.02% bromophenol blue, and 5% β-mercaptoethanol) with protease inhibitors, phosphatase inhibitors, and 1% phenylmethysulphonyl fluoride. Total protein was separated by electrophoresis on a 10% SDS-polyacrylamide gel, and protein bands were transferred onto a polyvinylidene difluoride membrane. The membrane was then incubated with primary antibodies at 4°C overnight. Antibody concentration ratios are listed in Supplementary Table 1. Subsequently, the membrane was incubated with horseradish peroxidase-conjugated anti-rabbit or anti-mouse secondary antibody for 1 hour at room temperature or 4°C overnight. Chemiluminescence was detected by Immobilon ECL Ultra Western HRP Substrate (Darmstadt Germany, Millipore).

### Immunofluorescence staining

Motile sperm were washed and resuspended at a concentration of 10 × 10^6^ cells/mL in PBS. A drop of 20 μL of sperm suspension was smeared onto a slide and allowed to air dry at room temperature. These slides were then fixed in 4% paraformaldehyde for 10 min and washed with PBS three times for 5 min each time. The sperm slides were subsequently perforated by 0.3% Triton X-100 with 0.2% Tween-20 in PBS for 30 min at room temperature. For antigen blocking, 3% BSA in 10% goat serum solution was used, followed by incubation at room temperature for 1 h. The slides were incubated with the primary antibody solution at a final concentration of 1:50 - 1:100 (Supplementary Table 1) at 4 °C overnight. After washing with PBST (PBS with 0.5% Tween-20) three times for 5 min each time, the samples were incubated with a polyclonal anti-rabbit/mouse IgG-FITC antibody (1:1000 dilution) for 1 h in the dark at room temperature. After three washes, the samples were then incubated with DAPI for 30 min at room temperature to colorize the nucleus. Finally, Vectashield® Antifade Mounting Medium (Vector Laboratories, CA, USA) was used to mount coverslips. Images were captured using a fluorescence confocal microscope (TCS SP8 SR, Leica, Germany).

### Untargeted metabolomics analysis

Sperm were treated in the absence or presence of 25 μM Bay117082 for 10 min, and then collected and purified. To remove proteins and extract metabolites, 800 μL cold methanol/acetonitrile (1:1, v/v) was added. This mixture was collected and centrifuged at 14000 *g* for 5 min at 4°C and the supernatant thus obtained was collected. After drying the supernatant in a vacuum centrifuge, it was redissolved in 100 μL acetonitrile/water (1:1, v/v) and subjected to LC-MS/MS. A quadrupole time-of-flight mass spectrometer (Sciex TripleTOF 6600, USA) coupled to hydrophilic interaction chromatography via electrospray ionization was used to analyze these extracts by Shanghai Applied Protein Technology Company Limited. LC separation was achieved on an ACQUIY UPLC BEH Amide column (2.1 mm×100 mm, 1.7 μm particle size) using an aqueous gradient of solvent A (25 mM ammonium acetate and 25 mM ammonium hydroxide) and solvent B (acetonitrile). Mass spectra were obtained in negative and positive ionizations mode. Data were acquired in the mass range from 60 to 1000 Da m/z range during MS acquisition, and the accumulation time for TOF MS scan was set at 0.20 s/spectra. Further, during auto-MS/MS acquisition, data were acquired in the mass range from 25 to 1000 Da m/z, and the accumulation time for product ion scan was set at 0.05 s/spectra. Information-dependent acquisition was used to acquire product ion scan in the high sensitivity mode.

### ATP and NADH/NAD^+^ level determination

Spermatozoa were treated with or without 25 μM Bay117082 for 10 min, and sperm were then collected and purified. Spermatozoa pellets were resuspended in lysis buffer from a kit and ultrasonically lysed (20 *kHz*, 750 W, 20% power, cycles of 2 s on and 5 s off for 1 min; Intelligent Ultrasonic Processor, SHUNMATECH, Nanjing, China) on ice. Sperm ATP levels were measured by the Enhanced ATP Assay Kit (Beyotime, S0027, China), according to manufacturer instructions. ATP concentration between 0.1 nM to 10 μM is proportional to the fluorescence when fluorescein and luciferase are extreme. Spermatozoa NADH/NAD^+^ levels were determined using the Coenzyme I NAD (H) content test kit (Nanjing Jiancheng Bioengineering Institute, A114-1-1, China). The oxidized thiazole blue (MTT) was reduced to formazan by NADH through the hydrogen transfer of phenazine methyl sulfate; the absorbance at 570 nm was determined, according to the manufacturer instructions. NAD^+^ is reduced to NADH by alcohol dehydrogenase and further detected by the MTT reduction method.

### Conditional knockout mouse model

Mice carrying a *LoxP-flanked Nfkbia* allele (*Nfkbia*^*flox/flox*^) were generated using the CRISPR-Cas9 technology and homologous recombination in fertilized eggs. *LoxP* sites were designed to be located at both ends of exons 1 and 2 of *Nfkbia*. Briefly, Cas9 mRNA and sgRNA (Supplementary Table 2) and donor vector were microinjected into the fertilized eggs of C57BL/6 J mice to obtain F0, which were then mated with C57BL/6 J mice and passaged to obtain a stable genetic (*Nfkbia*^*flox/flox*^) generation. *Nfkbia*^*flox/flox*^ females were mated with *Stra8-Cre* knock-in male mice expressing CRE from A1 spermatogonia onward^73^ (*Stra8-GFPCre* mice were generously provided by Prof. MingHan Tong) to generate (*Nfkbia*^*flox/-*^; *Stra8-Cre*) fitters and mate with (*Nfkbia*^*flox/flox*^) mice. Mice genotypically identified as (*Nfkbia*^*flox/flox*^; *Stra8-Cre*) were considered to be conditional knockout mice. Western blotting and immunofluorescence assays were performed to verify knockout efficiency.

### Statistics and reproducibility

Values represent mean ± standard error of the mean (SEM), with the number of samples (*n*) being ≥3 in independent experiments. GraphPad Prism 8.3 (Prism, USA) was used for data analysis. Data between the control and treatment groups were analyzed using two-tailed *t* tests, one-way ANOVA or two-way ANOVA to determine statistical significance. Tukey’s multiple comparison test was applied to analyze data for multiple comparisons. *p* < 0.05 indicated statistical significance. The statistical programming language R (www.R-project.org) and the web server for metabolomic data analysis MetaboAnalyst (https://www.metaboanalyst.ca) were used to analyze metabolomics data.

### Reporting summary

Further information on research design is available in the Nature Portfolio Reporting Summary linked to this article.

## Supplementary information

Supplementary Information

Description of Additional Supplementary Files

Supplementary Video 1

Supplementary Video 2

Supplementary Video 3

Supplementary Video 4

Supplementary Data 1

Reporting Summary

## Data Availability

All data analyzed during this study are included in this published article. The uncropped and unedited blots to make graphs are included in the Supplementary Figs. 3–8. The source data behind the graphs in the paper is provided in the Supplementary Data 1 file.
