# Supplementary material for: IKBA phosphorylation governs human sperm motility through ACC-mediated fatty acid beta-oxidation
Source: Commun Biol. 2023 Mar 25;6:323. doi: 10.1038/s42003-023-04693-6 (PMC10039860; doi:10.1038/s42003-023-04693-6)
Supplement: Supplementary file 1 — Supplementary Information [file 42003_2023_4693_MOESM1_ESM.pdf]

IKBA phosphorylation governs human sperm motility through ACC-mediated fatty acid beta-oxidation

Supplementary Information

**Supplementary Table 1.**	**2-3**
**Supplementary Table 2.**	**4**
**Supplementary Videos.**	**5**
**Supplementary Figure 1.**	**6-7**
**Supplementary Figure 2.**	**8**
**Supplementary Figure 3.**	**9**
**Supplementary Figure 4.**	**10**
**Supplementary Figure 5.**	**11**
**Supplementary Figure 6.**	**12**
**Supplementary Figure 7.**	**13**

Supplementary Table 1.

Antibodies Information				
**Antibodies**	**Manufactures**	**Product code**	**WB**	**IF**
Anti-IκB-α(C-21)	Santa Cruz Biotechnology	SC-371	1:1000	1:50
Anti-IκB-α(H-4)	Santa Cruz Biotechnology	sc-1643	1:1000	1:50
anti-IκB-β(C-20)	Santa Cruz Biotechnology	SC-945	1:1000	/
anti-IKKγ(B-3)	Santa Cruz Biotechnology	SC-8032	1:1000	/
Anti-phosphotyrosine monoclonal antibody	Merck Millipore Corporation	4G10	1:8000	/
anti-α-tubulin monoclonal antibody	Merck Millipore Corporation	T6199	1:10000	/
NF-κB p65 (D14E12) XP® Rabbit mAb	Cell Signaling Technology.	8242	1:2000	1:100
Phospho-NF-κB p65 (Ser536) (93H1) Rabbit mAb	Cell Signaling Technology.	3033	1:2000	/
RelB (D7D7W) Rabbit mAb	Cell Signaling Technology.	10544	1:2000	/
c-Rel (D4Y6M) Rabbit mAb	Cell Signaling Technology.	12707	1:2000	/
NF-κB1 p105/p50 (D4P4D) Rabbit mAb	Cell Signaling Technology.	13568	1:2000	/
NF-κB2 p100/p52 (D7A9K) Rabbit mAb	Cell Signaling Technology.	37359	1:2000	/
IKKα (3G12) Mouse mAb	Cell Signaling Technology.	61294	1:2000	1:100
IKKβ (D30C6) Rabbit mAb	Cell Signaling Technology.	8943	1:2000	1:100
Phospho-IKKα/β (Ser176/180) (16A6) Rabbit mAb	Cell Signaling Technology.	2697	1:2000	/
Phospho-IκBα (Ser32/36) (5A5) Mouse mAb	Cell Signaling Technology.	9246	1:2000	/
Acetyl-CoA Carboxylase (C83B10) Rabbit mAb	Cell Signaling Technology.	3676	1:2000	1:100

Phospho-Acetyl-CoA Carboxylase (Ser79) (D7D11) Rabbit mAb	Cell Signaling Technology.	11818	1:2000	/
Phospho-AMPKα (Thr172) (40H9) Rabbit mAb	Cell Signaling Technology.	2535	1:2000	/
AMPKα (D5A2) Rabbit mAb	Cell Signaling Technology.	5831	1:2000	/
anti-rabbit IgG horseradish peroxidase linked antibody	Cell Signaling Technology.	7074	1:5000	/
anti-mouse IgG horseradish peroxidase linked antibody	Cell Signaling Technology.	7076	1:5000	/
ACSL1 Polyclonal antibody	Proteintech	13989-1-AP	1:2000	1:100
ACADL-Specific Polyclonal antibody	Proteintech	17526-1-AP	1:2000	1:100
ACADVL Polyclonal Antibody	Proteintech	14527-1-AP	1:2000	1:100
HADHA Polyclonal antibody	Proteintech	10758-1-AP	1:2000	1:100
Anti-CPT1A antibody	Abcam	ab128568	1:2000	1:100
Donkey anti-Rabbit IgG (H+L) Highly Cross-Adsorbed Secondary Antibody, Alexa Fluor™ Plus 488	Thermo Scientific	A32790	/	1:1000
Donkey anti-Mouse IgG (H+L) Highly Cross-Adsorbed Secondary Antibody, Alexa Fluor™ Plus 488	Thermo Scientific	A32766	/	1:1000

Supplementary Table 2. sgRNAs used for generating knockout mice.

The sgRNAs targeting the gene's upstream (U) and downstream (D) regions are presented.

Gene		sgRNA sequences	PAM	KO/strategy
Nfkbia	U	TGAGACAGGGTTTACGTTGT	AGG	Zygote/Microinjection
	D	GAGGTCTTTATGACCCACCT	AGG	Zygote/Microinjection

Supplementary Videos

Supplementary Video 1 - 2. The motive video of sperm with relatively low VCL base level incubated in BWW medium with (Supplementary Video 1) or without (Supplementary Video 2) Bay117082 for 10 min. Sperm mainly displays as higher curvilinear velocity and an alteration from forward movement to curvilinear movement under the action of Bay117082.

Supplementary Video 3 - 4. The motive video of sperm with relatively high VCL base level incubated in BWW medium with (Supplementary Video 3) or without Bay117082 (Supplementary Video 4) for 10min. Sperm mainly displays an alteration from forward movement to curvilinear movement, but not a significantly increase of curvilinear velocity under the action of Bay117082.

Supplementary Figure 1

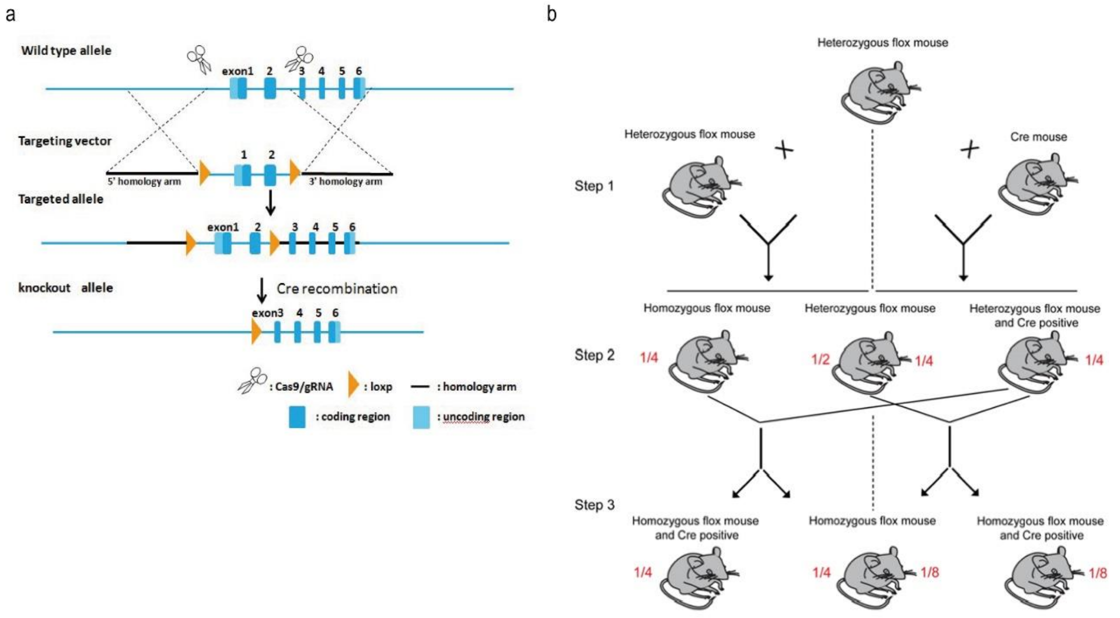

c

Gene		Primer	Size
Nfkbia	F	GGGGGCCTGGGTACATCTCTAAGT	WT: 379 bp; Heterozygous: 379 and 437 bp; Homozygous: 437 bp;
	R	ATCTGTGCTGGTGGGGTGGTTTGA	
Stra8-Cre	F	ACTCCAAGCACTGGGCAGAA	WT:702bp Mutant:400bp
	R1	GCCACCATAGCAGCATCAAA	
	R2	CGTTTACGTCGCCGTCCAG	

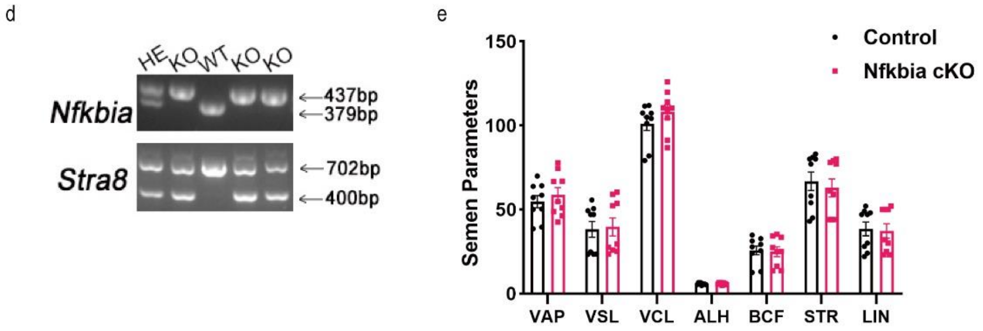

Supplementary Figure 1. Establishment of IKBA$^{-/-}$ mouse

a: Schematic diagram of *loxP-flanked Nfkbia* allele. We designed the *loxP* sites on both sides of exon1and exon2 of *Nfkbia* to make the deletion of IKBA protein.

b: The breeding strategy of IKBA conditional knockout mouse. Mice carrying *loxP-flanked Nfkbia* allele (*Nfkbia$^{flox/-}$*) crossed with *Stra8-Cre* mice. Fitters with genotype (*Nfkbia$^{flox/flox}$*; *Stra8-Cre*) were the IKBA spermatocyte conditional knockout mice.

c - d: Identification of mouse genotyping by PCR and the electrophoresis of PCR products was shown (d). The primers were indicated in the table (c). WT, wild type; HE, heterozygote; HO, homozygote.

e: Sperm motility parameters of control mouse and $IKBA^{-/-}$ mouse. n = 9.

Supplementary Figure 2.

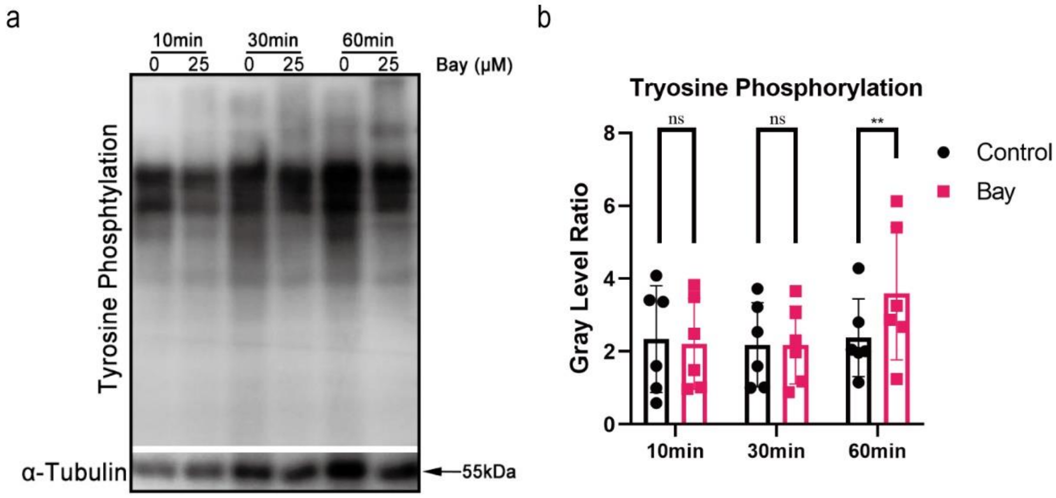

Supplementary Figure 2. The change of protein tyrosine phosphorylation level of sperm treated with Bay117082.

a: The protein tyrosine phosphorylation level of human sperm incubated in BWW with or without Bay117082 for 10, 30, and 60 mins. The antibody α-Tubulin was applied to assess protein loading.

b: Statistical analysis of gray values contained all bands visualized in protein tyrosine phosphorylation. n = 6. **$p < 0.01$ as compared with the corresponding control (0 μM).

Supplementary Figure 3

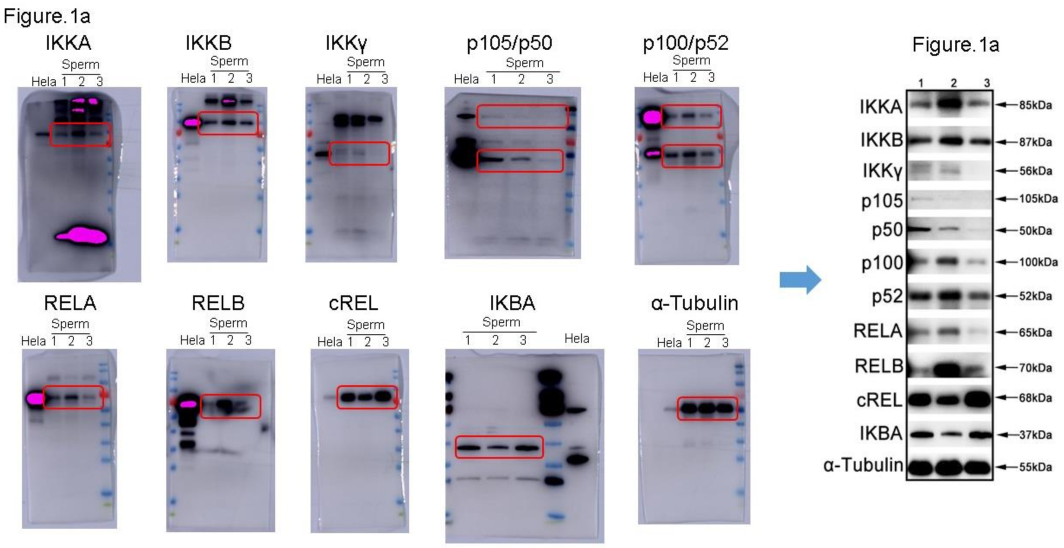

Supplementary Figure 3. The original images of blots of Fig. 1a

Supplementary Figure 4

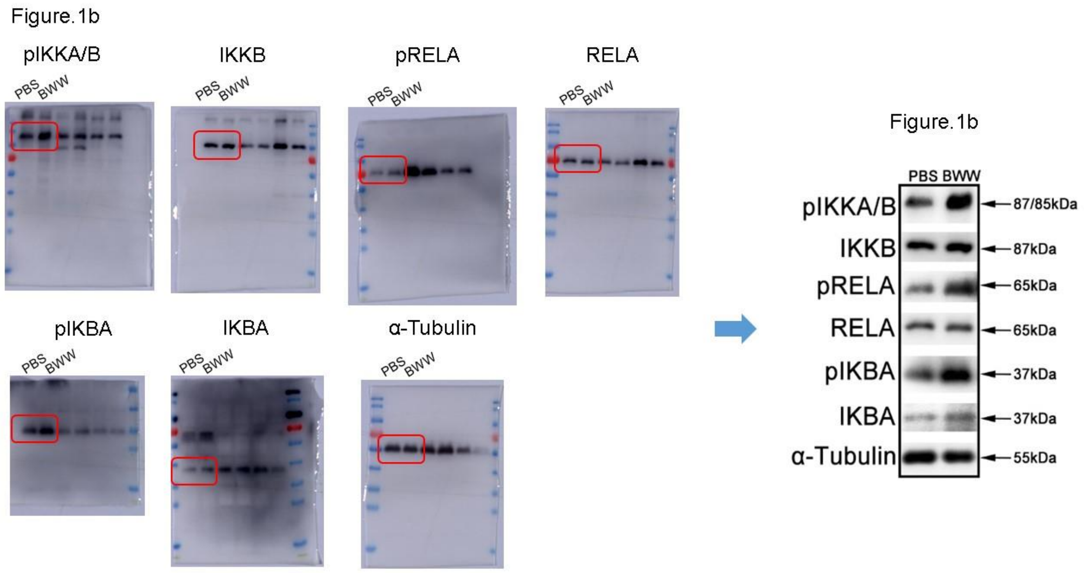

Supplementary Figure 4. The original images of blots of Fig. 1b

Supplementary Figure 5

Figure.2a

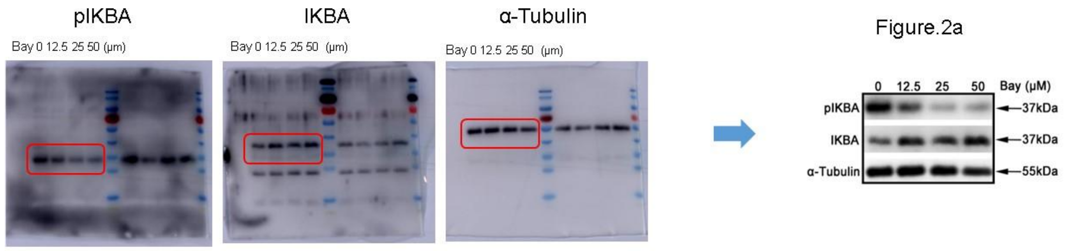

Figure.2d

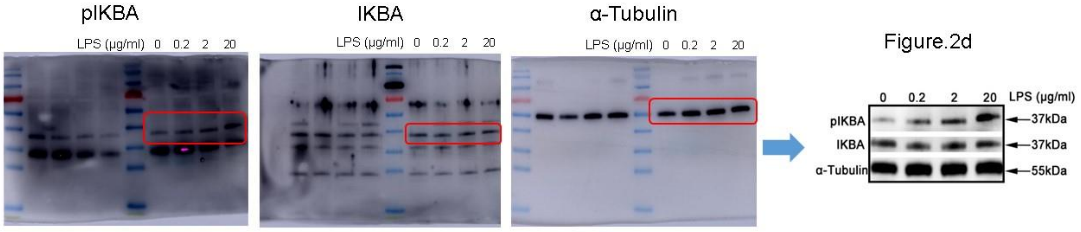

Supplementary Figure 5. The original images of blots of Fig. 2a and Fig. 2d

Supplementary Figure 6

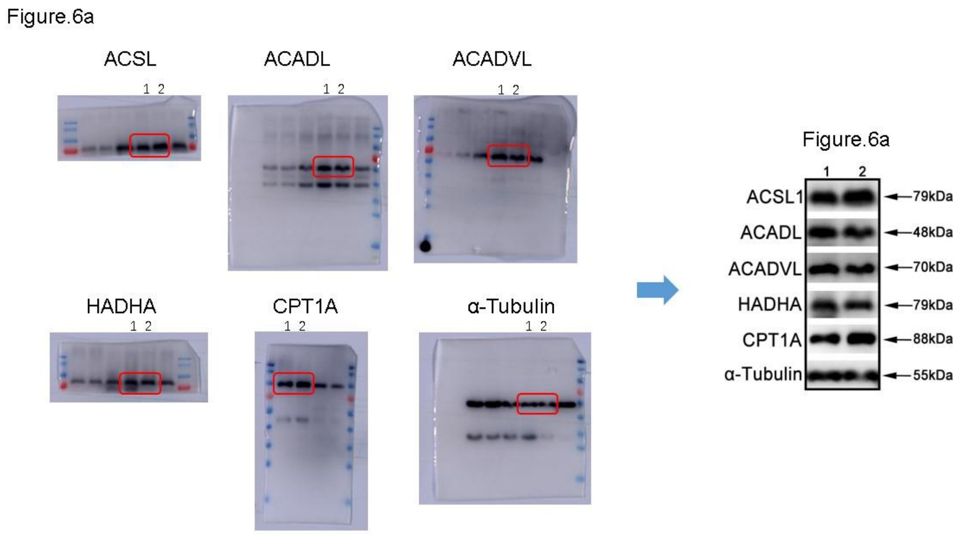

Supplementary Figure 6. The original images of blots of Fig. 6a

Supplementary Figure 7

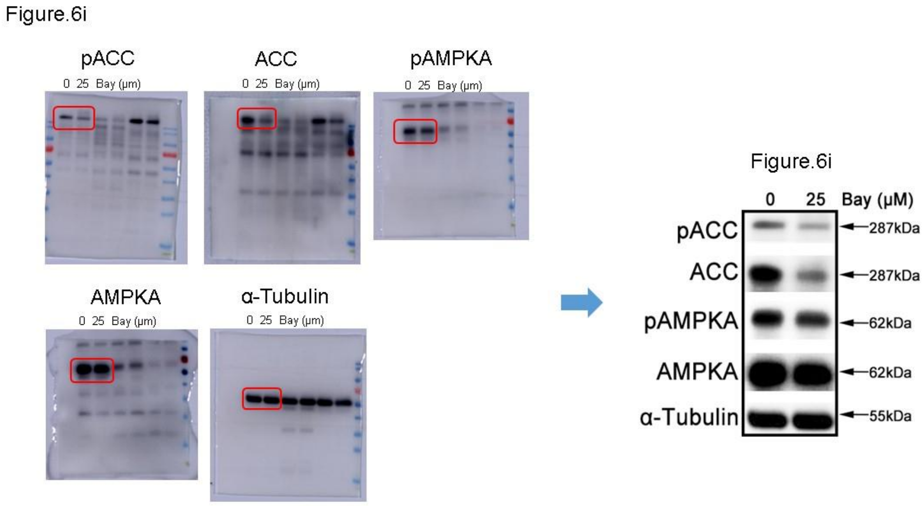

Supplementary Figure 7. The original images of blots of Fig. 6i

Supplementary Figure 8

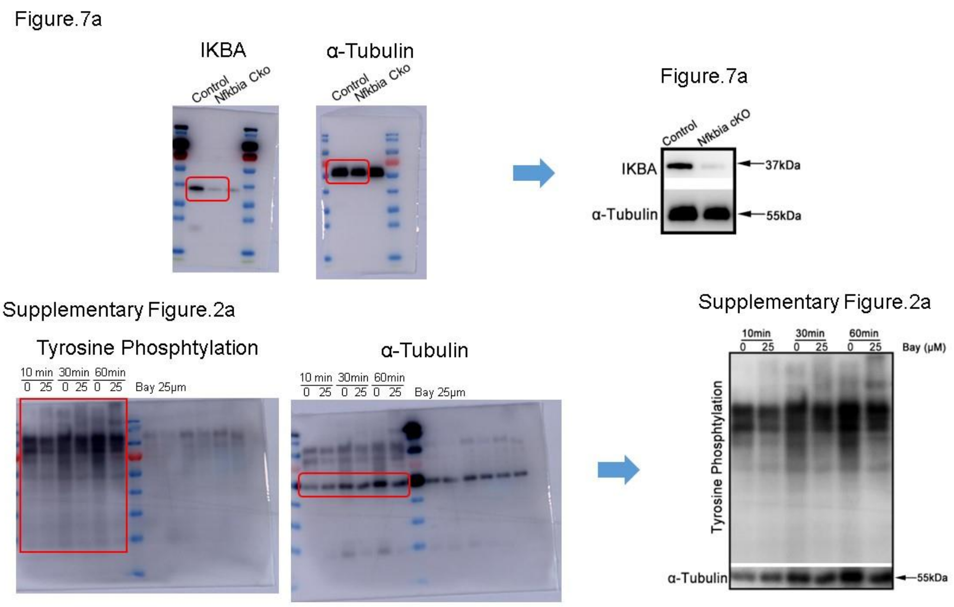

Supplementary Figure 8. The original images of blots of Fig. 7a and Supplementary Figure. 2a.
