# Supplementary material for: IKBA phosphorylation governs human sperm motility through ACC-mediated fatty acid beta-oxidation
Source: Commun Biol. 2023 Mar 25;6:323. doi: 10.1038/s42003-023-04693-6 (PMC10039860; doi:10.1038/s42003-023-04693-6)
Supplement: Supplementary file 2 — Description of Additional Supplementary Files [file 42003_2023_4693_MOESM2_ESM.pdf]

Description of Additional Supplementary Files

File Name: Supplementary Data 1

Description: The source data behind the graphs in the paper.

File Name: Supplementary Video 1

Description: The motive video of sperm with relatively low VCL base level incubated in BWW medium for 10 min.

File Name: Supplementary Video 2

Description: The motive video of sperm with relatively low VCL base level incubated in BWW medium with Bay117082 for 10 min.

File Name: Supplementary Video 3

Description: The motive video of sperm with relatively high VCL base level incubated in BWW medium for 10 min.

File Name: Supplementary Video 4

Description: The motive video of sperm with relatively high VCL base level incubated in BWW medium with Bay117082 for 10 min.
