# Supplementary material for: IKBA phosphorylation governs human sperm motility through ACC-mediated fatty acid beta-oxidation
Source: Commun Biol. 2023 Mar 25;6:323. doi: 10.1038/s42003-023-04693-6 (PMC10039860; doi:10.1038/s42003-023-04693-6)
Supplement: Supplementary file 8 — Reporting Summary [file 42003_2023_4693_MOESM8_ESM.pdf]

nature portfolio

Corresponding author(s): DBPR-COMMSBIO-22-4101B

Last updated by author(s): Feb 23, 2023

Reporting Summary

Nature Portfolio wishes to improve the reproducibility of the work that we publish. This form provides structure for consistency and transparency in reporting. For further information on Nature Portfolio policies, see our Editorial Policies and the Editorial Policy Checklist.

Statistics

For all statistical analyses, confirm that the following items are present in the figure legend, table legend, main text, or Methods section.

n/a	Confirmed	
☐	☒	The exact sample size (*n*) for each experimental group/condition, given as a discrete number and unit of measurement
☐	☒	A statement on whether measurements were taken from distinct samples or whether the same sample was measured repeatedly
☐	☒	The statistical test(s) used AND whether they are one- or two-sided<br>*Only common tests should be described solely by name; describe more complex techniques in the Methods section.*
☒	☐	A description of all covariates tested
☐	☒	A description of any assumptions or corrections, such as tests of normality and adjustment for multiple comparisons
☐	☒	A full description of the statistical parameters including central tendency (e.g. means) or other basic estimates (e.g. regression coefficient) AND variation (e.g. standard deviation) or associated estimates of uncertainty (e.g. confidence intervals)
☐	☒	For null hypothesis testing, the test statistic (e.g. F, t, r) with confidence intervals, effect sizes, degrees of freedom and P value noted<br>*Give P values as exact values whenever suitable.*
☒	☐	For Bayesian analysis, information on the choice of priors and Markov chain Monte Carlo settings
☐	☒	For hierarchical and complex designs, identification of the appropriate level for tests and full reporting of outcomes
☒	☐	Estimates of effect sizes (e.g. Cohen's *d*, Pearson's *r*), indicating how they were calculated

Our web collection on statistics for biologists contains articles on many of the points above.

Software and code

Policy information about availability of computer code

Data collection	OR
Data analysis	The online software MetaboAnalyst 5.0 were used for analysis of the untargeted metabolism state (https://www.metaboanalyst.ca). Metabolite Set Enrichment Analysis of metabolites based on The Small Molecule Pathway Database (https://smpdb.ca).

For manuscripts utilizing custom algorithms or software that are central to the research but not yet described in published literature, software must be made available to editors and reviewers. We strongly encourage code deposition in a community repository (e.g. GitHub). See the Nature Portfolio guidelines for submitting code & software for further information.

Data

Policy information about availability of data

All manuscripts must include a data availability statement. This statement should provide the following information, where applicable:

- Accession codes, unique identifiers, or web links for publicly available datasets
- A description of any restrictions on data availability
- For clinical datasets or third party data, please ensure that the statement adheres to our policy

Data available on request from the authors.

Human research participants

Policy information about studies involving human research participants and Sex and Gender in Research.

Reporting on sex and gender	*Use the terms sex (biological attribute) and gender (shaped by social and cultural circumstances) carefully in order to avoid confusing both terms. Indicate if findings apply to only one sex or gender; describe whether sex and gender were considered in study design whether sex and/or gender was determined based on self-reporting or assigned and methods used. Provide in the source data disaggregated sex and gender data where this information has been collected, and consent has been obtained for sharing of individual-level data; provide overall numbers in this Reporting Summary. Please state if this information has not been collected. Report sex- and gender-based analyses where performed, justify reasons for lack of sex- and gender-based analysis.*
Population characteristics	*Describe the covariate-relevant population characteristics of the human research participants (e.g. age, genotypic information, past and current diagnosis and treatment categories). If you filled out the behavioural & social sciences study design questions and have nothing to add here, write "See above."*
Recruitment	*Describe how participants were recruited. Outline any potential self-selection bias or other biases that may be present and how these are likely to impact results.*
Ethics oversight	*Identify the organization(s) that approved the study protocol.*

Note that full information on the approval of the study protocol must also be provided in the manuscript.

Field-specific reporting

Please select the one below that is the best fit for your research. If you are not sure, read the appropriate sections before making your selection.

☒ Life sciences ☐ Behavioural & social sciences ☐ Ecological, evolutionary & environmental sciences

For a reference copy of the document with all sections, see nature.com/documents/nr-reporting-summary-flat.pdf

Life sciences study design

All studies must disclose on these points even when the disclosure is negative.

Sample size	Most experiments were designed as paired test that one sample were divided into two or more equal parts and deal with different treatments. For pharmacological blocking tests and metabolites detection, at least 6 samples were used for the experiments and the significance were calculated to acquired the result. For the untargeted metabolomics analysis, 20 samples were used.
Data exclusions	All sperm samples involved in this research were classed as normozoospermic and asthenozoospermia according to World Health Organization (WHO) standards. Both normozoospermic and asthenozoospermia were used for Bay117082 validity. While only normal sperm were used to study the mechanism.
Replication	All experiments were replicated three times or more to confirm the conclusions.
Randomization	The specimens were randomly subjected to each experimental subgroup. The conditional knockout mice were grouped according to their genotype and identification results.
Blinding	Blinding were not used in our experimens because the concentration and dosage of a specific reagent or the genotype should be specified before the drug treatment experiment begins.

Reporting for specific materials, systems and methods

We require information from authors about some types of materials, experimental systems and methods used in many studies. Here, indicate whether each material, system or method listed is relevant to your study. If you are not sure if a list item applies to your research, read the appropriate section before selecting a response.

Materials & experimental systems

n/a	Involved in the study
☐	☒ Antibodies
☒	☐ Eukaryotic cell lines
☒	☐ Palaeontology and archaeology
☐	☒ Animals and other organisms
☒	☐ Clinical data
☒	☐ Dual use research of concern

Methods

n/a	Involved in the study
☒	☐ ChIP-seq
☒	☐ Flow cytometry
☒	☐ MRI-based neuroimaging

Antibodies

Antibodies used

Anti-IκB-α(C-21):SC-371, anti-IκB-α(H-4):sc-1643, anti-IκB-β(C-20): SC-945, anti-IKKγ(B-3): SC-8032 were purchased from Santa Cruz Biotechnology (Inc, Europe). Anti-phosphotyrosine monoclonal antibody 4G10, anti-α-tubulin monoclonal antibody:T6199 were purchased from Merck Millipore Corporation (Billerica, MA, USA). NF-κB p65 (D14E12) XP Rabbit mAb, Phospho-NF-κB p65 (Ser536) (93H1) Rabbit mAb, RelB (D7D7W) Rabbit mAb, c-Rel (D4Y6M) Rabbit mAb, NF-κB1 p105/p50 (D4P4D) Rabbit mAb, NF-κB2 p100/p52 (D7A9K) Rabbit mAb, IKKα (3G12) Mouse mAb, IKKβ (D30C6) Rabbit mAb, Phospho-IKKα/β (Ser176/180) (16A6) Rabbit mAb, Phospho-IκBα (Ser32/36) (5A5) Mouse mAb , Acetyl-CoA Carboxylase (C83B10) Rabbit mAb 3676, Phospho-Acetyl-CoA Carboxylase (Ser79) (D7D11) Rabbit mAb 11818, Phospho-AMPKα (Thr172) (40H9) Rabbit mAb 2535, AMPKα (D5A2) Rabbit mAb 5831, anti-rabbit IgG horseradish peroxidase linked antibody, anti-mouse IgG horseradish peroxidase linked antibody, were purchased from Cell Signaling Technology Corporation (Danvers, MA, USA). ACSL1 Polyclonal antibody (13989-1-AP), ACADL-Specific Polyclonal antibody :17526-1-AP, ACADVL Polyclonal Antibody:14527-1-AP, HADHA Polyclonal antibody: 10758-1-AP were purchsed from Proteintech. Anti-CPT1A antibody (ab128568) was purchsed from Abcam. Donkey anti-Rabbit IgG (H+L) Highly Cross-Adsorbed Secondary Antibody, Alexa Fluor™ Plus 488: A32790, Donkey anti-Mouse IgG (H+L) Highly Cross-Adsorbed Secondary Antibody, Alexa Fluor™ Plus 488: A32766 were purchsed from Thermo Scientific.

Validation

Validation statements of antibodies were applied on the manufacturer's website.

Animals and other research organisms

Policy information about studies involving animals; ARRIVE guidelines recommended for reporting animal research, and Sex and Gender in Research

Laboratory animals

C57BL/6J mice were used for the animals experiments as wild type. Mice carrying LoxP-flanked Nfkbia allele (Nfkbiaflox/flox) were generated by means of CRISPR-Cas9 technology and homologous recombination in fertilized eggs. The LoxP sites were designed to be located at both ends of exon 1 and exon 2 of Nfkbia. Stra8-GFPCre mice were generously provided by Prof. MingHan Tong. (Nfkbiaflox/flox) female mate with Stra8-Cre knock-in male mice to generate (Nfkbiaflox/flox; Stra8-Cre) mice that expected as conditional knockout mice. All mice were used at the age of 3-4 Month.

Wild animals

OR

Reporting on sex

All sperm were collected from male mice.

Field-collected samples

OR

Ethics oversight

Ethical approval for this study including the collection of semen samples was granted by Ethics Committee on human subjects of International Peace Maternity and Child Health Hospital (GKLW2018-03)

Note that full information on the approval of the study protocol must also be provided in the manuscript.
